# The relation between the incidence of hypernatremia and mortality in patients with severe traumatic brain injury

**DOI:** 10.1186/cc7953

**Published:** 2009-07-07

**Authors:** Umberto Maggiore, Edoardo Picetti, Elio Antonucci, Elisabetta Parenti, Giuseppe Regolisti, Mario Mergoni, Antonella Vezzani, Aderville Cabassi, Enrico Fiaccadori

**Affiliations:** 1 Dipartimento di Clinica Medica, Nefrologia & Scienze della Prevenzione, Universita' degli Studi di Parma, Via Gramsci 14, 43100 Parma, Italy; 2 1° Servizio di Anestesia & Rianimazione, Azienda Ospedaliera-Universitaria di Parma, Via Abbeveratoia 4, 43100 Parma, Italy

## Abstract

**Introduction:**

The study was aimed at verifying whether the occurrence of hypernatremia during the intensive care unit (ICU) stay increases the risk of death in patients with severe traumatic brain injury (TBI). We performed a retrospective study on a prospectively collected database including all patients consecutively admitted over a 3-year period with a diagnosis of TBI (post-resuscitation Glasgow Coma Score ≤ 8) to a general/neurotrauma ICU of a university hospital, providing critical care services in a catchment area of about 1,200,000 inhabitants.

**Methods:**

Demographic, clinical, and ICU laboratory data were prospectively collected; serum sodium was assessed an average of three times per day. Hypernatremia was defined as two daily values of serum sodium above 145 mmol/l. The major outcome was death in the ICU after 14 days. Cox proportional-hazards regression models were used, with time-dependent variates designed to reflect exposure over time during the ICU stay: hypernatremia, desmopressin acetate (DDAVP) administration as a surrogate marker for the presence of central diabetes insipidus, and urinary output. The same models were adjusted for potential confounding factors.

**Results:**

We included in the study 130 TBI patients (mean age 52 years (standard deviation 23); males 74%; median Glasgow Coma Score 3 (range 3 to 8); mean Simplified Acute Physiology Score II 50 (standard deviation 15)); all were mechanically ventilated; 35 (26.9%) died within 14 days after ICU admission. Hypernatremia was detected in 51.5% of the patients and in 15.9% of the 1,103 patient-day ICU follow-up. In most instances hypernatremia was mild (mean 150 mmol/l, interquartile range 148 to 152). The occurrence of hypernatremia was highest (*P *= 0.003) in patients with suspected central diabetes insipidus (25/130, 19.2%), a condition that was associated with increased severity of brain injury and ICU mortality. After adjustment for the baseline risk, the incidence of hypernatremia over the course of the ICU stay was significantly related with increased mortality (hazard ratio 3.00 (95% confidence interval: 1.34 to 6.51; *P *= 0.003)). However, DDAVP use modified this relation (*P *= 0.06), hypernatremia providing no additional prognostic information in the instances of suspected central diabetes insipidus.

**Conclusions:**

Mild hypernatremia is associated with an increased risk of death in patients with severe TBI. In a proportion of the patients the association between hypernatremia and death is accounted for by the presence of central diabetes insipidus.

## Introduction

Hypernatremia, a water balance disorder encountered in about 6 to 9% of critically ill patients, has been associated with an increased risk of death and complications in some recent retrospective studies in general intensive care units (ICUs) [1-3].

Patients with severe traumatic brain injury (TBI) have a high risk of developing hypernatremia over the course of their ICU stay, due to the coexistence of predisposing conditions such as impaired sensorium, altered thirst, central diabetes insipidus (CDI) with polyuria, and increased insensible losses [4]. Moreover, these patients often receive mannitol or hypertonic saline solutions, with the aim of reducing cerebral edema and controlling intracranial pressure [5]. In this clinical setting, it is not known, however, whether increased serum sodium (Na) is an independent risk factor for death, or is simply a surrogate marker of illness severity.

It has been shown that almost 20% of patients with subarachnoid hemorrhage develop hypernatremia, a complication bearing an increased risk of death [6]. On the other hand, in a recent series of patients from a neuro-ICU, hypernatremia was documented in only 8% of them; moreover, only the more advanced forms of this disorder (that is, serum Na exceeding 160 mmol/l) were associated with increased mortality [7]. These conflicting findings leave the question of the true clinical significance of moderate increases in serum Na (for example, between 145 and 160 mmol/l) unresolved.

We therefore designed the present study in order to verify whether the occurrence of hypernatremia during the ICU stay is an independent risk factor of death in patients with severe TBI (Glasgow Coma Score ≤ 8).

## Materials and methods

### Study population

We studied all adult patients consecutively admitted with a diagnosis of severe TBI from May 2004 to April 2006. The operational definition of severe TBI was a post-resuscitation Glasgow Coma Score of 8 or less at ICU admission.

The ICU of the Anesthesia and Intensive Care Department is located in part of the 1,200-bed Parma University Medical School Hospital, a tertiary academic referral institution. The ICU contains 20 general intensive care beds, staffed with full-time intensive care specialists. The unit provides all critical care services to patients admitted to the Emergency Department for head injury with or without polytrauma, as well as postoperative care for the neurosurgery services. The same ICU serves as a neurotrauma ICU for a catchment area of about 1,200,000 inhabitants.

### Data collection

Regarding the TBI patients admitted to the ICU, we prospectively collected data concerning demography, clinical and laboratory characteristics, prognostic factors and outcome, which were entered into an electronic database. For each patient the following data were obtained at admission: age, sex, cause of admission classified by type of trauma, premorbid functional status, acute and chronic co-morbidities, brain CT-scan data, Simplified Acute Physiology Score II score [8], Injury Severity Score [9], Glasgow Coma Score [10], hemodynamics, respiratory status and mechanical ventilation, blood gases, serum electrolytes, serum glucose, hemoglobin, leukocyte and platelet counts, renal function, and urinary output. Additional data were collected on a daily basis: serum electrolyte levels (all values, if more than one value was available), serum glucose, administered medications and fluids, including vasopressin and osmotic therapy (defined as the use of 3% or 5% saline or mannitol to treat cerebral edema or raised intracranial pressure), urinary volume, mechanical ventilation, and intracranial pressure (ICP) when available. The use of desmopressin acetate (DDAVP) was taken as a surrogate marker of suspected CDI. Finally, data concerning ICU complications, ICU mortality and inhospital mortality were also collected.

All subjects received standard care for TBI according to current guidelines [11,12]. The protocol dictated that routine clinical practice would never change for the purpose of study data collection. The Ethical Committee of the Parma University Medical School approved the study and waived the need for written informed consent by patients' next of kin.

### Generation of variates and missing values

Some clinical parameters were assessed hourly, and other parameters were assessed every 4 hours, 6 hours, 8 hours or once daily. Serum Na was assessed an average of three times a day. The number of determinations, however, tended to decrease with the increase in length of the ICU stay. To simplify the analysis, we created variates referring to the day of stay as the fundamental time unit.

We adopted three indexes to define the presence of serum Na disorders – daily serum Na, daily urinary output (polyuria being the marker of renal water loss), and daily administration of DDAVP.

Urinary output was the least reliable of these three indexes, as it was frequently missing. Some of the patients did not have complete (that is, 24-hour) urine output recorded. This problem occurred more frequently on the day that the most severely ill patients were admitted (in fact, missing urine output was significantly and independently associated with increased mortality; data not shown). In some other cases, exact urine output recording was missing during the hospital stay because the patients received intermittent urinary catheterization. Finally, urinary output was influenced by DDAVP medication, which the doctors administered whenever they noted an increase in urinary output (usually, an abrupt increase of urinary output to more than 250 ml/hour for 2 hours, in the absence of diuretic therapy), with the result of curbing the increased urinary output.

At variance with urinary output, there were only nine missing values regarding serum Na and no missing values concerning DDAVP use.

For the purpose of the analysis, the presence of hypernatremia was expressed as a time-dependent indicator variate. Hypernatremia was defined as serum Na >145 mmol/l on at least two occasions during 1 day of ICU stay. In 35% of the cases there was only a single daily determination, although this occurred for the most part during the second week of stay. The nine missing daily Na measurements were replaced by the value of the previous day of the ICU stay.

The use of DDAVP, which we took as a surrogate marker for the presence of CDI, was defined by a time-dependent indicator variate. To avoid the possibility that DDAVP could be interpreted as a marker of established brain death rather than a death predictor, the coding of the variate switched from 0 to 1 starting from the day after the first DDAVP administration.

We also created time-dependent indicator variates for the presence of daily urinary output above 3 l and for the use of mannitol and hypertonic saline solutions, and created time-dependent continuous variates for glucose levels and hyperglycemia (two daily serum glucose values above 10 mmol/l).

### Data analysis

We used Stata Release 10 software (2007; StataCorp, College Station, TX, USA) for all analyses.

#### Fourteen-day mortality

With the use of Cox proportional-hazards regression models, we examined the relation between 14-day ICU mortality and hypernatremia, polyuria (defined as urinary output >3 l day), and the use of DDAVP (that is, presence of CDI) over the course of the ICU stay. In order to adjust the estimates for the baseline risk of death, we used the core + CT score from the International Mission for Prognosis and Clinical Trial (IMPACT) prognostic model [13]. This score takes into account the extension of brain injury detected by CT scan at admission. Additionally, we adjusted the models for common determinants of polyuria (use of hypertonic Na solutions, intravenous mannitol, hyperglycemia), which may also be potentially associated with increased mortality in this category of patients.

In the principal analyses, patients were censored at the time of discharge. In a further analysis, all patients discharged from the ICU before day 14 were considered as surviving beyond day 14, with the exception of the patient who died at day 12 after discharge from the ICU. The covariate status after discharge from the ICU was not known, thus the last covariate before discharge was carried forward until the day of censoring or death. We do not report the results of these analyses because they were virtually identical to those of the main analyses.

We examined linearity of the continuous variates by the residual-based plots [14]. We tested departures from the proportional assumption using the procedure proposed by Grambsch and Therneau based on Shoenfeld residuals [15]. We used the Efron method to handle tied failures, the likelihood ratio test to compute *P *values, the profile likelihood for the point estimate and 95% confidence intervals [16].

We also decided to estimate the relation between hypernatremia and death after having stratified the data according to the presence of suspected CDI (that is, DDAVP use). With this aim in mind we fitted an interaction term between DDAVP use and hypernatremia in a stratified Cox regression model where DDAVP use was included as the stratum variable. To gain deeper insight into the nature of the observed relation between hypernatremia and mortality in the presence of CDI, we computed a measurement to explain variation in survival time (namely, *R*^2^), which is appropriate for use with the unstratified Cox regression models with time-dependent covariates. For this purpose we used the *strph2 *program, which computes Rosyton's modification of O'Quingley, Xu and Stare's modification of Nagelkerke's coefficient of determination for survival models [17,18].

We also compared the models with the Bayes information criterion. The model with the smallest value of the Bayes information criterion was considered better. The Bayes information criterion is a likelihood-based measure of fit, which adds a penalty for added covariates based on sample size. It seeks to balance the competing desire of finding the best model (in terms of maximizing the likelihood) with model parsimony (only including those covariates that significantly contribute to the model). For the computation of the Bayes information criterion we considered the sample size to be equal to 130 (that is, the number of patients).

#### Other analyses

Two-sample comparisons were performed by the *t *test or the Mann–Whitney test for the continuous variates, and by Fisher's exact test for the categorical variates. Mixed models (with patients fitted at random) were used for two-sample comparisons in the presence of repeated measurements. The variates were log-transformed whenever appropriate to improve normality. The within-subject association between the incidence of hypernatremia and DDAVP administration was examined with exact conditional logistic regression (with the patient fitted as the stratum variable). The between-subject cross-sectional association between DDAVP and hypernatremia (with the 130 patients classified according to the occurrence, at any time during the ICU stay, of hypernatremia or DDAVP administration) was examined with exact unconditional logistic regression. All reported *P *values are two tailed.

## Results

### Clinical characteristic of the study population, follow-up and mortality

We enrolled 130 patients with severe TBI. The characteristics of the population in our study are summarized in Table 1. All patients were mechanically ventilated, about one-half of them by tracheostomy. Only 52 patients (40%) suffered from an isolated TBI, while about one-half of the others also had thoracic trauma with lung involvement. A relevant proportion of the patients had skull fracture, brain contusion, or subarachnoid hemorrhage. Thirty-two patients had no pupillary light reflex at admission. CT scan at admission showed cerebral swelling with a midline shift in one-quarter of the patients (median shift, 10 mm) and cerebral herniation in about one-sixth of them. Forty-one percent underwent neurosurgical emergent procedures after admission to the ICU. Only 5% of the patients were severely hypotensive at admission, but about one-half of them required vasopressor administration during their ICU stay.

**Table 1:** Clinical and demographic characteristics at intensive care unit admission

Age (years)	51.8 (23)
Male gender	96 (74%)
Injury Severity Score	30.3 (7.7)
Simplified Acute Physiology Score II Score	49.8 (14.6)
Glasgow Coma Score	3 (3 to 8)
Motor score	1 (1 to 5)
1	78 (60.0%)
2	11 (8.5%)
3	11 (8.5%)
4	6 (4.6%)
5	24 (18.5%)
Absence of pupillary reflex	
Both	21 (16.2%)
One	11 (8.5%)
Systolic arterial pressure <90 mmHg	7 (5.4%)
Tracheal intubation	
Prehospital	105 (81.0%)
At admission	25 (19.2%)
Hypotension	16 (12.3%)
Diabetes	9 (6.9%)
History of heart disease	21 (16.2%)
History of arterial hypertension	24 (18.5%)
Chronic renal failure	1 (0.8%)
spO_2 _(pulse oxymetry) (%)	97.3 (5.7%)
Hypoxia	11 (8.5%)
Plasma HCO_3 _(mmol/l)	21.1 (3.4)
pCO_2 _(mmHg)	38.9 (7.7)
Midline shift on brain CT	32 (24.6%)
Cerebral edema on brain CT	31 (23.8%)
Cerebral herniation on brain CT	21 (16.2%)
Subarachnoid hemorrhage	62 (47.7%)
Epidural hematoma	19 (14.6%)
Presence of petechial hemorrhages	15 (11.5%)
Subdural hematoma	72 (55.4%)
Cerebral contusion	67 (51.5%)
Obliteration of the third ventricle or basal cisterns	31 (23.8%)
CT classification	
I	13 (10%)
II	6 (4.6%)
III/IV	9 (6.9%)
V/VI	102 (78.5%)
Urgent neurosurgery^a^	54 (41.5%)
Polytrauma	78 (60.0%)
Thoracic trauma	89 (68.5%)
Abdominal trauma	25 (29.2%)

Continuous variates presented as mean (standard deviation) or median (range); categorical variates presented as number (percentage). ^a^Within 4 hours after intensive care unit admission.

Of the 130 patients, 34 (26.2%) died in the ICU within 14 days after admission, after a total follow-up of 1,103 patient-days. One patient died on day 12 (that is, within 14 days after admission), when he had been already discharged from the ICU. Twenty-nine of the 34 deaths in the ICU occurred within 3 days after admission. Eleven patients were discharged from the ICU within 3 days, and another 42 patients were discharged between day 4 and day 14. The total inhospital mortality was 41/130 (31.5%).

### Hypernatremia during the ICU stay

The mean serum Na at admission was 139 mmol/l (standard deviation 3.9). Only three patients (2.3%) had serum Na above 145 mmol/l (maximum value 149 mmol/l). Altogether, 15.9% of the follow-up days were complicated by hypernatremia – occurring at least once in 51.5% of the patients for 31.0% of the duration of their stay in the ICU, even though it was mild. In fact, the highest serum Na in patients with hypernatremia was, on average, 150 mmol/l (range 146 to 164, interquartile range 148 to 152).

Urinary output was missing in 153 out of the 1,103 ICU days of follow-up. Unfortunately, the data on urinary output were not randomly missing. In fact, ICU mortality in patients with at least one missing urinary output was 51.2% (21/41), in comparison with 16.8% (15/89) in the remaining patients (*P *< 0.001). Polyuria was detected in 34.4% (327/950) of ICU days, and occurred in 76.0% of the 108 subjects in whom urinary output was recorded. In the instances of ascertained polyuria, the mean urinary output was 4,150 ml/day – the maximum being 8,850 ml/day.

Twenty-five patients (19.2%) received DDAVP at least once over the course of their ICU stay. DDAVP, however, was administered only during 5.9% of the days of the entire follow-up. Patients receiving DDAVP had a higher urinary output and serum Na than those not receiving this medication (median urinary output 3,720 vs. 2,480 ml/day, *P *< 0.001; median serum Na 148 vs. 142 mmol/l, *P *< 0.001). For each patient the probability of receiving DDAVP increased with the onset of hypernatremia (odds ratio = 3.41, *P *= 0.009 by conditional logistic regression). Accordingly, 29.9% (20/67) of the patients who developed hypernatremia at any time during their ICU stay received DDAVP, compared with 7.9% (5/63) of the others (odds ratio = 4.88, *P *= 0.003 by unconditional logistic regression).

**Figure 1:**
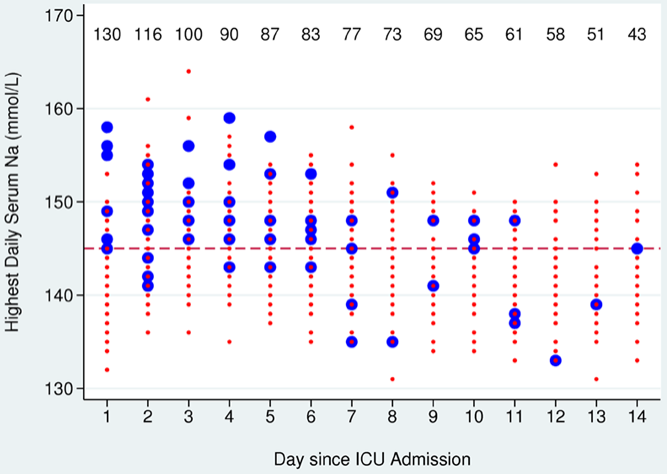
Highest daily serum sodium during the intensive care unit stay. Serum sodium (Na) values measured during intensive care unit (ICU) days when desmopressin acetate (DDAVP) was not administered (red dots) and when DDAVP was administered (blue circles). Data reported in the upper part of the plot represent the number of patients under observation on each ICU day of stay (the number decreases from left to right owing to discharge from ICU or owing to patient death). Horizontal dotted line, cut-off level of 145 mmol/l used to define hypernatremia. DDAVP was associated with higher serum Na levels (*P *< 0.001). The association between DDAVP and hypernatremia was not evident in the latest period of the ICU stay, possibly owing to the lowering effect of DDAVP on serum Na.

Figure 1 reports the crude data regarding the highest daily serum Na recorded over the patients' ICU stay. Serum Na is reported as a red dot or a blue circle according to whether DDAVP was administered on the same ICU day of stay. The relation between DDAVP and high serum Na was not evident during the final days of ICU stay, possibly owing to the lowering effect of DDAVP on serum Na.

Overall, these analyses suggest that DDAVP was, in fact, used whenever the physician in charge of the patient's care suspected CDI. Notably, the administration of DDAVP during the ICU stay was also associated with severe brain injury at admission (data not shown).

Mannitol was administered in 49.2% (64/130) of the patients over 27.9% (308/1,103) of the days spent in the ICU. Hypertonic saline solutions were administered in 36.1% (47/130) patients and over 14.3% (158/1,103) of the days they spent in the ICU. These interventions did not bear any apparent relation to serum Na or DDAVP administration (data not shown). The 51 patients in whom the concomitant measurement of serum Na and ICP was available did not show any difference in ICP according to the presence of hypernatremia (median ICP 16 mmHg in both instances, *P *= 0.67).

The average of the daily mean serum glucose was 7.7 mmol/l (range 3.3 to 17.0). Hyperglycemia occurred at least once in 37.7% (46/130) of the patients during 7.7% (85/1,103) days of stay in the ICU. There was no significant difference in mean glucose levels and in the rate of hyperglycemia according to DDAVP use or the presence of hypernatremia (data not shown).

### Relation between hypernatremia and ICU mortality

Patients who died on days 2 and 3 of their ICU stay had the highest increase in daily average serum Na between days 1 and 2, while receiving DDAVP more often than the others. In fact, the 13 patients who died on day 2 had a mean increase of serum Na of +3.7 mmol/l, which was higher than that observed in the same period in the 103 patients still alive in the ICU on day 2 (+1.5 mmol/l; *P *= 0.020). The mean increase in the four patients who died on day 3 was +4.6 mmol/l; that is, greater than that observed in the 96 patients who were still alive in the ICU on day 3 (+1.4 mmol/l; *P *= 0.019). Accordingly, patients who died on days 2 and 3 had received DDAVP more frequently than those who remained alive in the ICU. In fact, on day 2 the proportion of DDAVP use was 3/13 (23.1%) among patients who died and was 3/103 (2.9%) among those who were still alive (*P *= 0.018). On day 3, this proportion of DDAVP use was 3/4 (75.0%) and 4/96 (4.2%), respectively (*P *= 0.001). Overall, 56% (14/25) of the patients who received DDAVP at any time during their ICU stay died, compared with 19.0% (20/105) of the others (*P *= 0.001).

These findings were mirrored by the results of Cox proportional-hazards regression analysis. As shown in Table 2, hypernatremia was associated with a threefold increase in the hazard of ICU death even after adjustment for baseline risk (hazard ratio = 3.00 (95% confidence interval: 1.34 to 6.51; *P *= 0.003)). The additional adjustment for DDAVP use, however, halved the estimated relative increase in mortality (hazard ratio of hypernatremia adjusted for DDAVP use = 2.04; *P *= 0.092). On the other hand, after adjustment for hypernatremia, the hazard ratio associated with DDAVP use was 3.88 (*P *= 0.005) (Table 2). The *R*^2 ^values of the model that included the baseline risk were 0.543, 0.596, and 0.624 for hypernatremia, for DDAVP, and for hypernatremia + DDAVP, respectively. As shown in Table 2, after stratifying the model according to DDAVP use (that is, presence of suspected CDI), hypernatremia did not bear any additional prognostic information in the presence of CDI (hazard ratio = 0.58; *P *= 0.57), while retaining its importance in the other instances (hazard ratio = 4.20; *P *= 0.004) (*P *= 0.060 for the test of the difference between the two hazard ratios).

**Table 2:** Disorder of water balance over the course of the ICU stay and ICU mortality

Variate	Hazard ratio	95% confidence interval	*P *value	Bayes information criterion^a^
Crude analysis				
Hypernatremia	3.34	1.55 to 6.88	0.002	313.29
DDAVP use	8.23	3.24 to 19.52	<0.001	304.75
Adjusted for baseline risk of death				
Hypernatremia	3.00	1.34 to 6.51	0.003	291.17
DDAVP use	5.48	2.13 to 13.21	<0.001	286.07
Adjusted for baseline risk of death and for each other				
Hypernatremia	2.04	0.81 to 4.84	0.092	288.09
DDAVP use	3.88	1.40 to 10.33	0.005	
Hypernatremia adjusted for baseline risk and stratified according to DDAVP use				
Hypernatremia with DDAVP use	0.58	0.07 to 3.67	0.57	256.53
Hypernatremia without DDAVP use	4.20	1.62 to 10.17	0.004	

Hazard ratio, 95% confidence intervals, and *P *values associated with hypernatremia and desmopressin acetate (DDAVP) use in the intensive care unit (ICU), estimated by six different Cox proportional-hazards regression models. Hazard ratios associated with hypernatremia and DDAVP use were first estimated in separate models, without adjusting for confounding factors (Crude analysis), and after adjusting for baseline risk (Adjusted for baseline risk of death). They were then estimated including hypernatremia and DDAVP use in the same model in order to isolate the effect of each variate independently of the other (Adjusted for baseline risk of death and for each other). Finally, the hazard ratio associated with hypernatremia was estimated stratifying the Cox regression model for DDAVP use (Hypernatremia adjusted for baseline risk and stratified according to DDAVP use). Baseline risk is represented by the score from the International Mission for Prognosis and Analysis CT prognostic model [11]. ^a^A measure of both model fit and parsimony; the better the model, the smaller the associated BIC value.

Additional adjustment for the use of mannitol, hypertonic saline solution and hyperglycemia did not change the findings (data not shown). In fact the latter, which was associated with increased mortality, was evenly distributed according to the presence of hypernatremia and the use of DDAVP (data not shown).

## Discussion

To our knowledge, the present study is the first that has been specifically aimed at investigating the incidence and clinical significance of hypernatremia occurring during the course of the ICU stay in a large series of patients with severe TBI. The study shows that, in the immediate post-TBI period, mild hypernatremia is associated with an increased risk of death – although, in a proportion of the patients, this association is due to the occurrence of CDI, a marker of the extension and severity of brain injury.

We acknowledge that our study has the significant weakness of using DDAVP as the major criterion for diagnosing CDI, which, at best, can be considered a surrogate index only. Agha and colleagues defined CDI in the immediate post-TBI as serum Na >145 mmol/l in the presence of both polyuria (>3.5 l/day) and diluted urine (osmolality < 300 mOsm/l) [19,20]. We could not use the same criteria for the diagnosis of CDI, in as much as data on urinary output were missing in many instances and urine osmolality was not measured. Our analyses, however, showed a CDI incidence of 19.2% (25/130); that is, well within the range of 15 to 26% documented by the previous studies on the subject [19-21]. This concordant finding suggests that in our series CDI was correctly classified. In another small series of TBI patients the incidence of CDI was much lower [22], probably owing to the exclusion of patients with incomplete data. Similarly to those studies, we found that CDI is associated with an increase in the severity of brain injury [19] and in the risk of death [21,22]. Finally, our analysis was adjusted for several factors potentially capable of confounding the relation between hypernatremia, CDI and mortality; namely, the use of hypertonic saline solution, intravenous mannitol, serum glucose levels, and the incidence of hyperglycemia [23-25].

The high incidence of CDI that both we and other workers found [19-21] is not unexpected in patients with TBI [26]. The awareness of the importance of CDI is such that a decade ago a small randomized controlled study was designed to evaluate whether or not the use of DDAVP in *all *brain-dead donors (by definition, in patients with the most severe degree of brain injury) could improve kidney transplant function [27,28]. Injury of the hypothalamus and pituitary generally occurs concomitantly, and is seen at autopsy in up to 60% of patients dying from head trauma [22]. Edwards and Clark reviewed a series of pathological studies of fatal head injury and reported that hemorrhage or infarction in the hypothalamus was detected in 42% of cases [29]. The petechial hemorrhage areas in the anterior hypothalamic nuclei and neurohypophysis can be caused by forces transmitted to the head on impact, by increased ICP resulting from the brain edema, by shearing stresses that produce disruption of the pituitary stalk, and by the hypothalamic–hypophyseal portal system [30].

Our results confirm the recent finding from Hadjizacharia and colleagues that CDI is an independent risk indicator of death [21]. In fact, in our study the presence of CDI provided additional prognostic information regarding the extension of brain injury with respect to the CT scan at admission, because the relative hazard of mortality associated with CDI was adjusted for the CT IMPACT prognostic model as assessed at ICU admission. We found that the incidence of hypernatremia (occurring in about one-half of the patients at any time during the ICU stay, with 16% of ICU days complicated by this sodium disorder) was more than double the incidence of CDI. This incidence is higher than that reported by Qureshi and colleagues (19%) [6] and by Wartenberg and colleagues (22%) [31,32]. The latter two series, however, included patients with subarachnoid hemorrhage rather than with TBI; moreover, the study by Qureshi and colleagues defined hypernatremia by serum Na at admission or on day 3, and the study by Wartenberg defined hypernatremia as serum Na >150 mmol/l. Another study from a very large database (The Traumatic Coma Data Bank) reported an occurrence of electrolyte abnormalities in patients affected by TBI as high as 59%, with a peak incidence in the first 24 to 96 hours [33]; unfortunately, the true incidence of hypernatremia cannot be inferred from the data presented in the study, as all types of electrolyte disturbances were pooled together.

To our knowledge, ours is the first study documenting the incidence of hypernatremia during the ICU stay in severe TBI patients. The definition of hypernatremia in our study refers to the first 14 days of ICU stay, and it is robust since it requires that at least two values of serum Na be >145 mmol/l in all patients receiving multiple daily determinations of serum sodium. The finding that the incidence of CDI was lower than that of hypernatremia suggests that only a minority of the cases of hypernatremia were due to CDI. In most cases hypernatremia was generally mild, probably because the prompt administration of DDAVP by the attending physician prevented excess water loss if CDI was present. Van Beek and colleagues recently examined the relation between serum Na and outcome using data from the IMPACT database [34]. Their analysis took into consideration only serum Na values at admission, however, not those obtained during the ICU stay. At variance with what is observed during the ICU stay, patients with TBI show hypernatremia only rarely at admission, which in fact was detected only in 5% of the patients of the IMPACT study and in 2.3% of the patients in our study. In that setting Van Beek and colleagues defined high serum Na as Na levels above the 75th percentile, corresponding to 142 mmol/l [34]; that is, a level lower than the standard cut-off value currently used for defining hypernatremia.

Our findings indicate that in a proportion of the patients the relation between hypernatremia and mortality is accounted for by the coexistence of CDI, whereas hypernatremia by itself could represent an independent risk factor of death in those patients lacking CDI. We recognize that our criteria for assessing CDI might have identified only its full blown forms, however, possibly leaving undetected those incomplete and subtle forms that still can cause hypernatremia; this might explain the residual relation we found between hypernatremia and death. Further studies are needed to provide support for this hypothesis.

Finally, the relation between hypernatremia and mortality has been already documented in studies mostly dealing with patients in general ICUs [1-3,35,36], and not specifically including TBI patients. Even on the basis of the more recent literature, unfortunately based on retrospective studies only [1-3], it is not however possible to definitely exclude the possibility that hypernatremia in the ICU could simply be regarded as a surrogate marker of illness severity, rather than as an independent predictor of mortality. In the case of patients with TBI the interpretation of the relation between high serum Na levels and outcome is made even more difficult by the presence of peculiar interfering factors – such as for example CDI, as previously discussed – and the use of hypertonic saline to control cerebral edema and elevated ICP [5,33,37-43]. Hypertonic saline has actually gained major interest as a treatment option in patients with elevated ICP levels due to a wide spectrum of etiologies, such as subarachnoid hemorrhage [44-47], stroke [48,49], elective brain surgery [50], as well as other clinical conditions characterized by cerebral edema [51-53]. The proposed mechanisms of hypertonic saline action are complex, involving cell volume reduction due to fluid drawing from the brain, reduced cerebral blood volume due to ameliorated blood viscosity and rheology, greater neuroprotection through the restoring of neuronal membrane potentials, neuroinflammatory pathway modulation, and so forth [54].

It is to be noted that most available data about hypertonic saline use (either as intravenous boluses or continuous infusion) in TBI patients with high ICP levels derive from small trials, case series or retrospective studies [55-59], while only few papers deal with its possible side effects. Following the recent publication of a retrospective analysis of neurocritically ill patients including severe TBI [59], some concern has been raised about the use of continuous-infusion hypertonic saline [54]. In that study, hypertonic saline use increased the risk of hypernatremia, increased the number of infection days, increased the hospital length of stay, increased the creatinine and blood urea nitrogen serum levels, along with increasing the occurrence of deep vein thrombosis – the most severe form (serum Na >160 mmol/l) being eventually associated with an increased mortality [59]. Clearly, before recommending such treatment in clinical practice [60], we strongly need randomized-control intervention studies to confirm the safety and efficacy of hypertonic saline in the care of neurocritically ill patients.

## Conclusions

Mild hypernatremia is frequently encountered in patients with severe TBI during the ICU stay. In this clinical setting, a proportion of the cases of hypernatremia is probably due to the onset of CDI – an independent marker of brain injury severity and an independent prognostic indicator of ICU death. Be this and/or other mechanisms at play, hypernatremia is anyhow independently related with an increased risk of death.

## Key messages

• Mild hypernatremia is frequently encountered in patients with severe TBI during the ICU stay.

• In this clinical setting, a proportion of the cases of hypernatremia are likely to be due to the onset of CDI – an independent marker of brain injury severity and an independent prognostic indicator of ICU death.

• This knowledge notwithstanding, hypernatremia is independently related with increased risk of death.

## Abbreviations

CDI: central diabetes insipidus; CT: computed tomography; DDAVP: desmopressin acetate; ICU: intensive care unit; ICP: intracranial pressure; IMPACT: International Mission for Prognosis and Analysis of Clinical Trials in TBI; Na: sodium; TBI: traumatic brain injury.

## Competing interests

The authors declare that they have no competing interests.

## Authors' contributions

EF, EPi and UM conceived of the study and participated in its design. MM, EA, EPa and AV coordinated the study. UM, GR and EF performed the statistical analysis. EF, UM and AC drafted the manuscript. All authors read and approved the final manuscript.

## References

[B1] Lindner G Funk GC Schwarz C Kneidinger N Kaider A Schneeweiss B Kramer L Druml W Hypernatremia in the critically ill is an independent risk factor for mortality Am J Kidney Dis 2007 50 952 957 10.1053/j.ajkd.2007.08.016 18037096

[B2] Hoorn EJ Betjes MGH Weigel J Zietse R Hypernatremia in critically ill patients: too little water and too much salt Nephrol Dial Transplant 2008 23 1562 1568 10.1093/ndt/gfm831 18065827

[B3] Stelfox HT Ahmed SB Khandwala F Zygun D Shahpori R Laupland K The epidemiology of intensive care unit acquired hyponatremia and hypernatremia in medical–surgical intensive care units Crit Care 2008 12 R162 19094227 10.1186/cc7162 PMC2646327

[B4] Tisdall M Crocker M Watkiss J Smith M Disturbances of sodium in critically ill adult neurologic patients: a clinical review J Neurosurg Anesthesiol 2006 18 57 63 16369141 10.1097/01.ana.0000191280.05170.0f PMC1513666

[B5] Peterson B Khanna S Fisher B Marshall L Prolonged hypernatremia controls elevated intracranial pressure in head-injured pediatric patients Crit Care Med 2000 28 1136 1143 10.1097/00003246-200004000-00037 10809295

[B6] Qureshi A Suri FK Sung GY Straw RN Yahia AM Saad M Guterman LR Hopkins LN Prognostic significance of hypernatremia and hyponatremia among patients with aneurismal subarachnoid hemorrhage Neurosurgery 2002 50 749 756 10.1097/00006123-200204000-00012 11904025

[B7] Aiyagary V Deibert E Diringer MN Hypernatremia in the neurologic intensive care unit: how high is too high? J Crit Care 2006 21 163 172 10.1016/j.jcrc.2005.10.002 16769461

[B8] Le Gall JR Lemeshow S Saulnier F A new Simplified Acute Physiology Score (SAPS II) based on a European/North American multicenter study JAMA 1993 270 2957 2963 10.1001/jama.270.24.2957 8254858

[B9] Baker SP O'Neill B Haddon W Jr Long WB The Injury Severity Score: a method for describing patients with multiple injuries and evaluating emergency care J Trauma 1974 14 187 196 10.1097/00005373-197403000-00001 4814394

[B10] Teasdale G Jennett B Assessment of coma and impaired consciousness: a practical scale Lancet 1974 2 81 82 10.1016/S0140-6736(74)91639-0 4136544

[B11] The Brain Trauma Foundation. The American Association of Neurological Surgeons. The Joint Section on Neurotrauma and Critical Care. Trauma systems J Neurotrauma 2000 17 457 627 10.1089/neu.2000.17.457 10937887

[B12] Brain Trauma Foundation, American Association of Neurological Surgeons, Joint Section in Neurotrauma and Critical care Guidelines for the Management of Severe Traumatic Brain Injury: Cerebral Perfusion Pressure. Updated CPP Guidelines 2003 Approved by the American Association of Neurological Surgeons; New York (NY): Brain Trauma Foundation, Inc

[B13] Steyerberg EW Mushkudiani N Perel P Butcher I Lu J McHugh GS Murray GD Marmarou A Roberts I Habbema JD Maas AI Predicting outcome after traumatic brain injury: development and international validation of prognostic scores based on admission characteristics PLoS Med 2008 5 e165 18684008 10.1371/journal.pmed.0050165 PMC2494563

[B14] Hosmer DW Lemeshow S Applied Survival Analysis 1999 New York: John Wiley & Sons

[B15] Grambsch PM Therneau TM Proportional hazard tests and diagnostics based on weighted residuals Biometrika 1994 81 515 526 10.1093/biomet/81.3.515

[B16] Royston P Explained variation for survival models Stata J 2006 6 83 96

[B17] O'Quigley J Xu R Stare J Explained randomness in proportional hazards models Stat Med 2005 24 479 489 10.1002/sim.1946 15532086

[B18] Royston P Profile likelihood for estimation and confidence intervals Stata J 2007 7 376 387

[B19] Agha A Thornton E O'Kelly P Tormey W Phillips J Thompson CJ Posterior pituitary dysfunction after traumatic brain injury J Clin Endocrinol Metab 2004 89 5987 5992 10.1210/jc.2004-1058 15579748

[B20] Agha A Sherlock M Phillips J Tormey W Thompson CJ The natural history of post-traumatic neurohypophysal dysfunction Eur J Endocrinol 2005 152 371 377 10.1530/eje.1.01861 15757853

[B21] Hadjizacharia P Beale EO Inaba K Chan LS Demetriades D Acute diabetes insipidus in severe head injury: a prospective study J Am Coll Surg 2008 207 477 484 18926448 10.1016/j.jamcollsurg.2008.04.017

[B22] Boughey JC Yost MJ Bynoe RP Diabetes insipidus in the head-injured patient Am Surg 2004 70 500 503 15212402

[B23] Yang SY Zhang S Wang ML Clinical significance of admission hyperglycemia and factors related to it in patients with acute severe head injury Surg Neurol 1995 44 373 377 10.1016/0090-3019(96)80243-6 8553258

[B24] Rovlias A Kotsou S The influence of hyperglycemia on neurological outcome in patients with severe head injury Neurosurgery 2000 46 335 343 10.1097/00006123-200002000-00015 10690722

[B25] Oddo M Schmidt JM Mayer SA Chiolero RL Glucose control after severe brain injury Curr Opin Clin Nutr Metab Care 2008 11 134 139 10.1097/MCO.0b013e3282f37b43 18301088

[B26] Schneider HJ Kreitschmann-Andermahar I Ghigo E Stalla GK Agha A Hypothalamopituitary dysfunction following traumatic brain injury and aneurismal subarachnoid hemorrhage JAMA 2007 298 1429 1438 10.1001/jama.298.12.1429 17895459

[B27] Phongsamran PV Critical care pharmacy in donor management Prog Transplant 2004 14 105 111 15264455 10.1177/152692480401400205

[B28] Guesde R Barrou B Leblanc I Ourahma S Goarin JP Coriat P Riou B Administration of desmopressin in brain-dead donors and renal function in kidney patients Lancet 1998 352 1178 1181 10.1016/S0140-6736(98)05456-7 9777834

[B29] Edwards OM Clark JD Posttraumatic hypopituitarism: six cases and review of the literature Medicine 1986 65 281 290 10.1097/00005792-198609000-00001 3018425

[B30] Kauffman HH Timberlake G Voelker J Pait TG Medical complications of head injury Med Clin North Am 1993 77 43 60 8419723 10.1016/s0025-7125(16)30271-1

[B31] Wartenberg KE Mayer SA Medical complications after subarachnoid hemorrhage: new strategies for prevention and management Curr Opin Crit Care 2006 12 78 84 10.1097/01.ccx.0000216571.80944.65 16543780

[B32] Wartenberg KE Schmidt JM Claassen J Temes RE Frontera JA Ostapkovich N Parra A Connolly ES Mayer SA Impact of medical complications on outcome after subharachnoid hemorrhage Crit Care Med 2006 34 617 623 10.1097/00003246-200612002-00426 16521258

[B33] Piek J Chesnut RM Marshall LF an Berkum-Clark M Klauber MR Blunt BA Eisenberg HM Jane JA Marmarou A Foulkes MA Extracranial complications of severe head injury J Neurosurg 1992 77 901 907 10.3171/jns.1992.77.6.0901 1432133

[B34] Van Beek JG Mushkudiani NA Steyerberg EW Butcher I McHugh GS Lu J Marmarou A Murray GD Maas AI Prognostic value of admission laboratory parameters in traumatic brain injury: results from the IMPACT study J Neurotrauma 2007 24 315 328 10.1089/neu.2006.0034 17375996

[B35] Polderman KH Schreuder WO Strack van Schijndel RJ Thijs LG Hypernatremia in the intensive care unit: an indicator of quality of care? Crit Care Med 1999 27 1041 1042 10.1097/00003246-199906000-00029 10397213

[B36] Kraft MD Btaiche IF Sacks GS Kudsk KA Treatment of electrolyte disorders in adult patients in the intensive care unit Am J Health Syst Pharm 2005 62 1663 1682 16085929 10.2146/ajhp040300

[B37] Valadka AB Robertson CS Should we be using hypertonic saline to treat intracranial hypertension? Crit Care Med 2000 28 1245 1246 10.1097/00003246-200004000-00069 10809326

[B38] Khanna S Davis D Peterson B Fisher B Tung H O'Quigley J Deutsch R Use of hypertonic saline in the treatment of severe refractory posttraumatic intracranial hypertension in pediatric traumatic brain injury Crit Care Med 2000 28 1144 1151 10.1097/00003246-200004000-00038 10809296

[B39] Marik PA Varon J Trask T Management of head trauma Chest 2002 122 699 711 10.1378/chest.122.2.699 12171853

[B40] Ogden AT Mayer SA Connolly ES Hyperosmolar agents in neurosurgical practice: the evolving role of hypertonic saline Neurosurgery 2005 57 207 215 10.1227/01.NEU.0000166533.79031.D8 16094147

[B41] Helmy A Vizcaychipi M Gupta AK Traumatic brain injury: intensive care management Br J Anaesth 2007 99 32 42 17556349 10.1093/bja/aem139

[B42] Petit L Masson F Cottenceau V Sztark F [Controlled hypernatremia] Ann Fr Anaesth Reanim 2006 25 828 837 16860968 10.1016/j.annfar.2006.04.005

[B43] Froelich M Hartl R Ultra-early hyperosmolar treatment in traumatic brain injury: will surgeons soon be old-school? Crit Care Med 2008 36 642 643 10.1097/CCM.0B013E3181629821 18216627

[B44] Horn P Munch E Vajkoczy P Herrmann P Quintel M Schilling L Schmiedek P Schürer L Hypertonic saline solution for control of elevated intracranial pressure in patients with exhausted response to mannitol and barbiturates Neurol Res 1999 21 758 764 10596385 10.1080/01616412.1999.11741010

[B45] Suarez JI Qureshi AI Bhardwaj A Williams MA Schnitzer MS Mirski M Hanley DF Ulatowski JA Treatment of refractory intracranial hypertension with 23.4% saline Crit Care Med 1998 26 1118 1122 10.1097/00003246-199806000-00038 9635664

[B46] Suarez JI Qureshi AI Parekh PD Razumovsky A Tamargo RJ Bhardwaj A Ulatowski JA Administration of hypertonic (3%) sodium chloride/acetate in hyponatremic patients with symptomatic vasospasm following subarachnoid hemorrhage J Neurosurg Anesthesiol 1999 11 178 184 10.1097/00008506-199907000-00004 10414672

[B47] Tseng MY Al-Rawi PG Czosnyka M Smielewski P Diehl RR Pickard JD Czosnyka M Enhancement of cerebral blood flow using systemic hypertonic saline therapy improves outcome in patients with poor-grade spontaneous subarachnoid hemorrhage J Neurosurg 2007 107 274 282 10.3171/JNS-07/08/0274 17695380

[B48] Schwarz S Georgiadis D Aschoff A Schwab S Effects of hypertonic (10%) saline in patients with raised intracranial pressure after stroke Stroke 2002 33 136 140 10.1161/hs0102.100877 11779902

[B49] Schwarz S Schwab S Bertram M Aschoff A Hacke W Effects of hypertonic saline hydroxyethyl starch solution and mannitol in patients with increased intracranial pressure after stroke Stroke 1998 29 1550 1555 9707191 10.1161/01.str.29.8.1550

[B50] Gemma M Cozzi S Tommasino C Mungo M Calvi MR Cipriani A Garancini MP 7.5% hypertonic saline versus 20% mannitol during elective neurosurgical supratentorial procedures J Neurosurg Anesthesiol 1997 9 329 334 10.1097/00008506-199710000-00007 9339405

[B51] Detry O De Roover A Honore P Meurisse M Brain edema and intracranial hypertension in fulminant hepatic failure: pathophysiology and management World J Gastroenterol 2006 12 7405 7412 17167826 10.3748/wjg.v12.i46.7405 PMC4087583

[B52] Murphy N Auzinger G Berdel W Wendon J The effect of hypertonic sodium chloride on intracranial pressure in patients with acute liver failure Hepatology 2004 39 464 470 10.1002/hep.20056 14767999

[B53] Raghavan M Marik PE Therapy of intracranial hypertension in patients with fulminant hepatic failure Neurocrit Care 2006 4 179 189 10.1385/NCC:4:2:179 16627910

[B54] Muizelaar JP Shahlaie K Hypertonic saline in neurocritical care: is continuous infusion appropriate? Crit Care Med 2009 37 1521 1523 10.1097/CCM.0b013e31819d3ea0 19318850

[B55] Brain Trauma Foundation; American Association of Neurological Surgeons; Congress of Neurological Surgeons; Joint Section on Neurotrauma and Critical Care, AANS/CNS Bratton SL Chestnut RM Ghajar J McConnell Hammond FF Harris OA Hartl R Manley GT Nemecek A Newell DW Rosenthal G Schouten J Shutter L Timmons SD Ullman JS Videtta W Wilberger JE Wright DW Guidelines for the management of severe traumatic brain injury. II. Hyperosmolar therapy J Neurotrauma 2007 24 Suppl 1 S14 S20 17511539 10.1089/neu.2007.9994

[B56] Hartl R Ghajar J Hochleuthner H Mauritz W Hypertonic/hyperoncotic saline reliably reduces ICP in severely head-injured patients with intracranial hypertension Acta Neurochir Suppl 1997 70 126 129 9416299 10.1007/978-3-7091-6837-0_39

[B57] Munar F Ferrer AM de Nadal M Poca MA Pedraza S Sahuquillo J Garnacho A Cerebral hemodynamic effects of 7.2% hypertonic saline in patients with head injury and raised intracranial pressure J Neurotrauma 2000 17 41 51 10.1089/neu.2000.17.41 10674757

[B58] Vialet R Albanese J Thomachot L Antonini F Bourgouin A Alliez B Martin C Isovolume hypertonic solutes (sodium chloride or mannitol) in the treatment of refractory posttraumatic intracranial hypertension: 2 mL/kg 7.5% saline is more effective than 2 mL/kg 20% mannitol Crit Care Med 2003 31 1683 1687 10.1097/01.CCM.0000063268.91710.DF 12794404

[B59] Froelich M Quanhong N Wess C Ougorets I Hartl R Continuous hypertonic saline therapy and the occurrence of complications in neurocritically ill patients Crit Care Med 2009 37 1433 1441 10.1097/CCM.0b013e31819c1933 19242317

[B60] Forsyth LL Liu-DeRyke X Parker D Jr Rhoney DH Role of hypertonic saline for the management of intracranial hypertension after stroke and traumatic brain injury Pharmacotherapy 2008 28 469 484 10.1592/phco.28.4.469 18363531

